# Case Report: MR-LINAC-guided adaptive radiotherapy for gastric cancer

**DOI:** 10.3389/fonc.2023.1159197

**Published:** 2023-09-08

**Authors:** Yajun Song, Yun Zhang, Huadong Wang, Mengyu Zhao, Fada Guan, Zhenjiang Li, Jinbo Yue

**Affiliations:** ^1^ Department of Graduate, Shandong First Medical University and Shandong Academy of Medical Sciences, Jinan, China; ^2^ Department of Radiation Oncology, Shandong Cancer Hospital and Institute, Shandong First Medical University and Shandong Academy of Medical Sciences, Jinan, China; ^3^ Department of Radiation Physics, Shandong Cancer Hospital and Institute, Shandong First Medical University and Shandong Academy of Medical Sciences, Jinan, China; ^4^ Cheeloo College of Medicine, Shandong University, Jinan, China; ^5^ Department of Therapeutic Radiology, Yale University School of Medicine, New Haven, CT, United States

**Keywords:** online adaptive radiation therapy, magnetic resonance guided radiation therapy, MR-LINAC, gastric cancer, adaptive planning, radiotherapy

## Abstract

**Background:**

The stomach is one of the most deformable organs. Its shape can be easily affected by breathing movements, and daily diet, and it also varies when the body position is different. The susceptibility of stomach has made it challenging to treat gastric cancer using the conventional image-guided radiotherapy, i.e., the techniques based on kilovoltage X-ray imaging. The magnetic resonance imaging guided radiotherapy (MRgRT) is usually implemented using a hybrid system MR-LINAC. It is feasible to implement adaptive radiotherapy using MR-LINAC for deformable organs such as stomach. In this case report, we present our clinical experience to treat a gastric cancer patient using MR-LINAC.

**Case description:**

The patient is a 58-year-old male who started having black stools with no apparent cause a year ago. Gastroscopy result showed pancreatic cancer, pathology: adenocarcinoma on gastric cancer biopsy, adenocarcinoma on gastric body minor curvature biopsy. The patient was diagnosed with gastric cancer (adenocarcinoma, cTxN+M1, stage IV, HER-2 positive). The patient was treated in 25 fractions with radiotherapy using MR-LINAC with online adaptive treatment plans daily. The target area in daily MR images varied considerably when compared with the target area on the CT simulation images. During the course of treatment, there have even been instances where the planned target area where the patient received radiotherapy did not cover the lesion of the day.

**Conclusion:**

Online adaptive MRgRT can be a meaningful innovation for treating malignancies in the upper abdomen. The results in the current study are promising and are indicative for further optimizing online adaptive MRgRT in patients with inoperable tumors of the upper abdomen.

## Introduction

Radiation therapy is one of the most effective treatments for stomach cancer (1). However, radiotherapy for gastric cancer is challenging owing to poor visualization of intraluminal tumors on computed tomography (CT) imaging, leading to geographic uncertainty and thus wide latitude for treatment planning. Radiotherapy for stomach tumors is limited by the proximity of surrounding organs with limited radiation tolerance, such as the duodenum, small intestine and liver: organs at risk (OAR) (2, 3). Stomach can move substantially during the respiratory cycle due to their proximity to the diaphragm. This is inevitably associated with increased doses to organs at risk, such as the duodenum and liver. Physiological movements (e.g., respiratory cycles) can misalign the target volume and OAR during treatment. Motion management is therefore critical in achieving safe and effective treatment. Using the four-dimensional CT (4DCT) simulation, which is performed at different phases in the respiratory cycle, allows for more accurate expansion of target volumes to reflect patient-specific breathing movements (4).

X-ray imaging methods such as kV radiography and cone beam CT (CBCT) are commonly used in image-guided radiation therapy (IGRT) for patient setup prior to treatment. This reduces the effects of potential setup errors caused by anatomical changes in target and OAR location (5). However, CBCT is relatively poorer than magnetic resonance imaging (MRI) in terms of soft tissue contrast. Therefore, CBCT is not suitable for imaging the abdomen region. As such, it is challenging to treat gastric cancer using the x-ray imaging based IGRT. To further improve image quality during radiation therapy, the concept of combining an MRI scanner with a linear accelerator has been proposed. Using this hybrid system, called MR-LINAC, has the potential to implement less toxic and more effective treatments in radiation oncology (6, 7). In recent years, MR-LINAC systems have been introduced into clinical applications. Using an MR-LINAC system allows for online adjustment of the treatment plan to compensate for anatomical changes between different treatment fractions. Literature has shown that using an MR-LINAC system improves the applicability and accuracy of radiation therapy for upper abdominal tumors (8), and in particular, it is more effective against tumors in deformable organs such as the stomach. In the currently reported case, we used a 1.5T MR-LINAC (Unity MR-LINAC, Elekta, Stockholm, Sweden) system to illustrate the MR-guided radiotherapy (MRgRT) implementation in treating gastric tumors through daily online adaptive plans. The Unity MR-LINAC system consists of a 7 MV linear accelerator and a 1.5 T MRI scanner. T2 weighted MR images were obtained using abdominal sequence parameters: TR = 2000 ms, TE = 206 ms, relative SNR = 1, ACQ matrix M×P = 232×167.

## Case description

The patient is a 58-year-old male who presents with a feeling of fullness in the upper abdomen after meal. The patient began to have black stools with no apparent cause in June 2021, several times a day. Gastroenteroscopy showed cardia cancer and diagnostic CT images showed localized thickening and enhancement of the gastric wall on the lesser curvature after treatment for gastric cancer. Pathology results were: adenocarcinoma on cardia biopsy, adenocarcinoma on biopsy of the lesser curvature of the gastric body, immunohistochemistry: Her-2 (3+), pMMR, PD-L1 expression CPS=0, EBER (–). Patient was diagnosed with gastric cancer (adenocarcinoma, cTxN+M1, stage IV, HER-2 positive).

The patient received concurrent chemoradiotherapy with oral tegafur-gimeracil-oteracil potassium (S-1) and 25 sessions of online adaptive MR-guided radiotherapy for gastric tumors. The patient was treated with the Unity MR-LINAC. The patient underwent a 4D CT scan with an empty stomach. Before CT simulation, anisodamine (dose 10 mg) was given to reduce gastric motility. The cardia and gastric body lesions (clinical target area volume, CTV) was outlined on the 4DCT scan and the images were combined into an internal CTV (ICTV). The combined ICTV included enlarged metastatic lymph nodes and a planned target area volume (PTV) extending 5 mm outwards from the ICTV. The patient was treated with involved site radiation therapy (ISRT)at 50 Gy in 25 fractions. Patient continued to be treated with Tislelizumab and Herceptin during this period. The patient fasted and received anisodamine (10 mg dose) prior to each radiation therapy session (**Figure S2**). As of September 2022, the patient’s follow-up CT results showed stable efficacy and no adverse symptoms such as nausea, vomiting, as well as abdominal pain.

**Figure 1:**
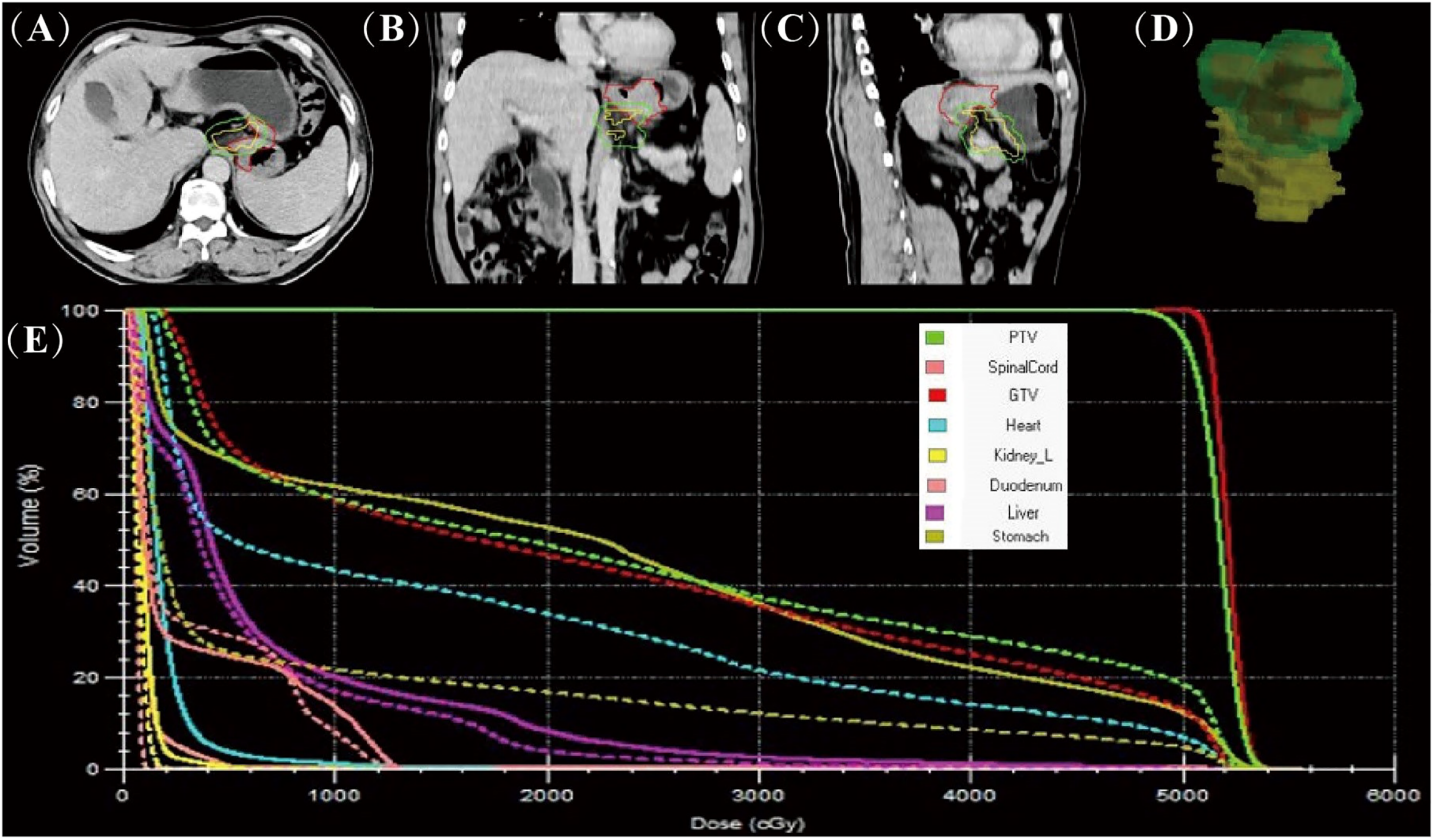
Comparison of online MR-guided radiotherapy planning and CT simulated localisation.The images are based on the CT simulated positioning, with the target areas of the first MRI positioning projected onto the images in axial **(A)**, coronal **(B)**, sagittal **(C)** and 3D stereoscopic structures **(D)** (red line delineates the stomach on the simulation images, the yellow line delineates the stomach on the daily MR images, and the green line delineates the stomach considering 1 cm external expansion from the ICTV). The accumulated DVH curves based on the online MR-guided radiotherapy plan (solid lines) and when this plan is used for CT simulated positioning (dotted lines) **(E)**.

Tumors were outlined based on artificial intelligence technology (AccuContour^®^) and the resulting images were then reviewed and modified by the attending radiation oncologist. The MR images of stomach tumors were clearer in patients taking scopolamine compared to those not taking it (**Figure S1**). After the online adaptive plan was approved by physicist and physician, the radiation treatment started with real-time MR imaging in all three planes to monitor the tumor and organs motion during the beam delivery. We found a significant difference between the patient’s first MRI-based guided radiotherapy and CT-based simulated localization of the target area outlined (**Figures 1A–D**). Using dose accumulation, there was a large cumulative dose difference between online MRI-guided radiotherapy and CT-simulated localization (**Figure 1E**). The cumulative DVH of the patient’s radiotherapy plan based on CT simulated localization showed a GTV50 of only D16%. The treatment would be ineffective if no online positioning was taken.

**Figure 2:**
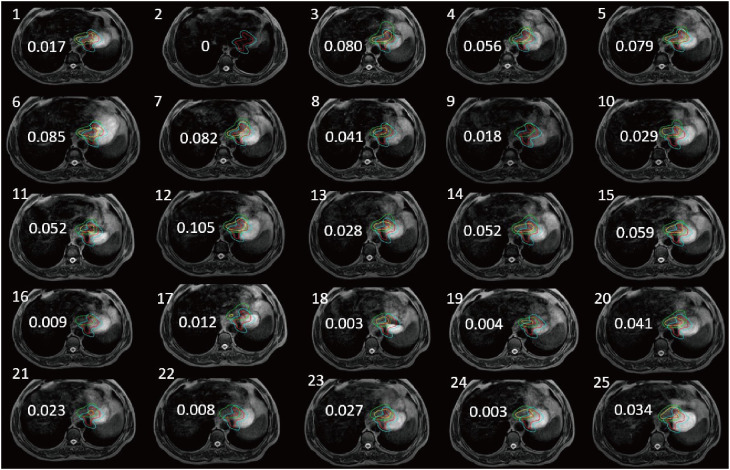
Tumors of the stomach delineation DSC (numerical value on each panel) of real-time MR imaging and simulation CT imaging (the red line delineates the stomach on the simulation images, the blue line delineates the stomach considering 5 mm external expansion from the ICTV, the yellow line delineates the stomach on the daily MRI images, and the green line delineates the stomach considering 5 mm external expansion from the ICTV).


The patient underwent 25 subsequent MRI-guided radiotherapy sessions. We calculated dice similarity coefficient (DSC) after outlining the gastric tumor lesions on real-time MR imaging and simulated CT imaging. With CBCT guidance, even after enlarging the PTV by 5 mm as usual, the tumor area was not completely covered at any fraction (**Figure 2**). The DSC measures the volumetric overlap of two sets of data and was obtained with Equation (1), which calculates the quotient of similarity between two volumetric sets with a value between 0 and 1. In this formula, A and B are the volumes of the target area outlined after CT-based localization and daily MRI-based localization, respectively. A DSC value of 1 means perfect overlap, whereas a DSC value of 0 means no overlap.

(1) $$DSC\left(A,B\right)=\frac{2\left|A\cap^{\;}B\right|}{\left|A\right|+\left|B\right|}$$

**Figure 3:**
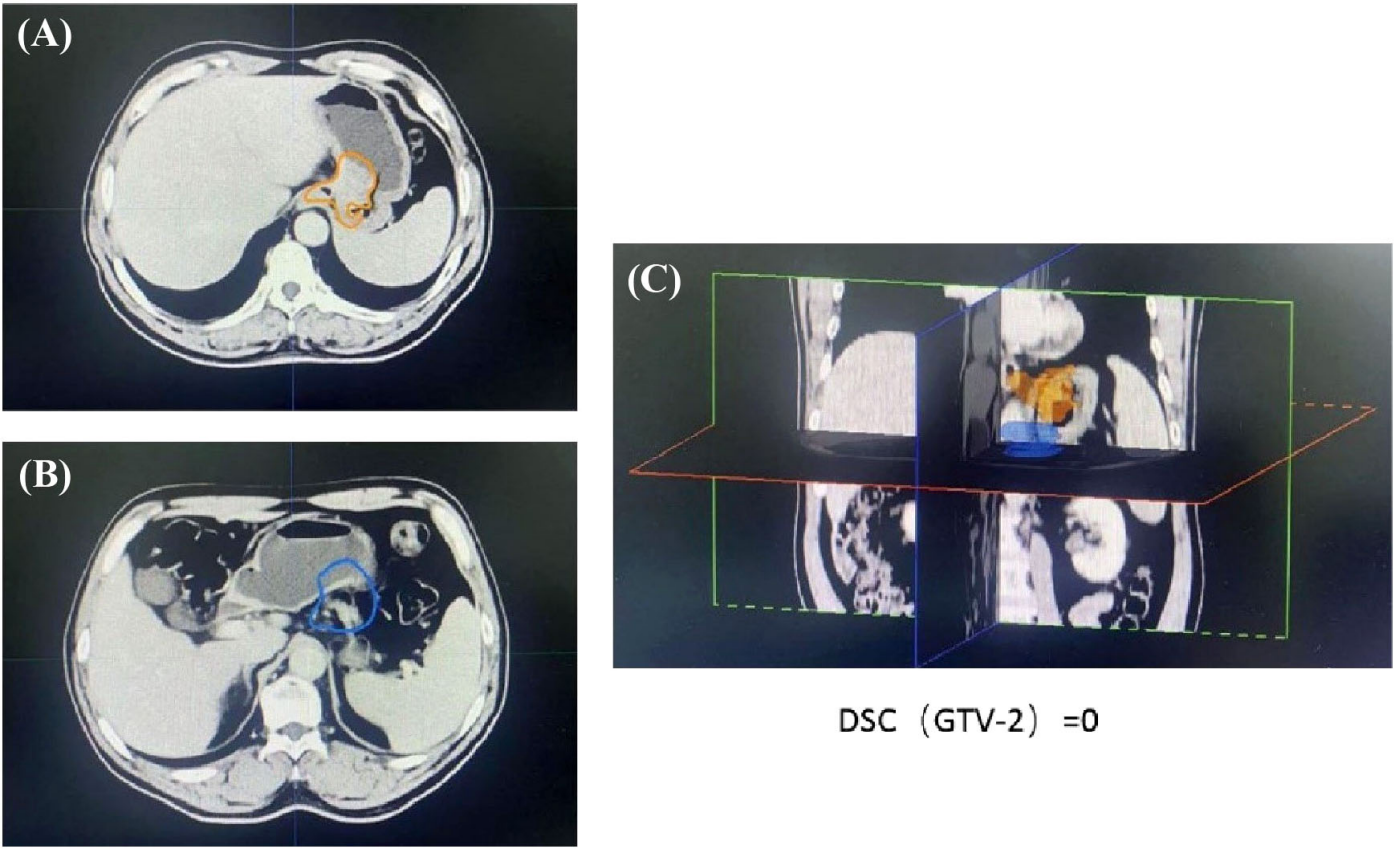
The images from the second treatment fraction with a DSC of 0 for the target area localized by MRI and the target area localized by simulated CT. (The orange line **(A, C)** delineates the stomach on the CT simulation images, the blue line **(B, C)** delineates the stomach on the daily MRI images).

**Figure 4:**
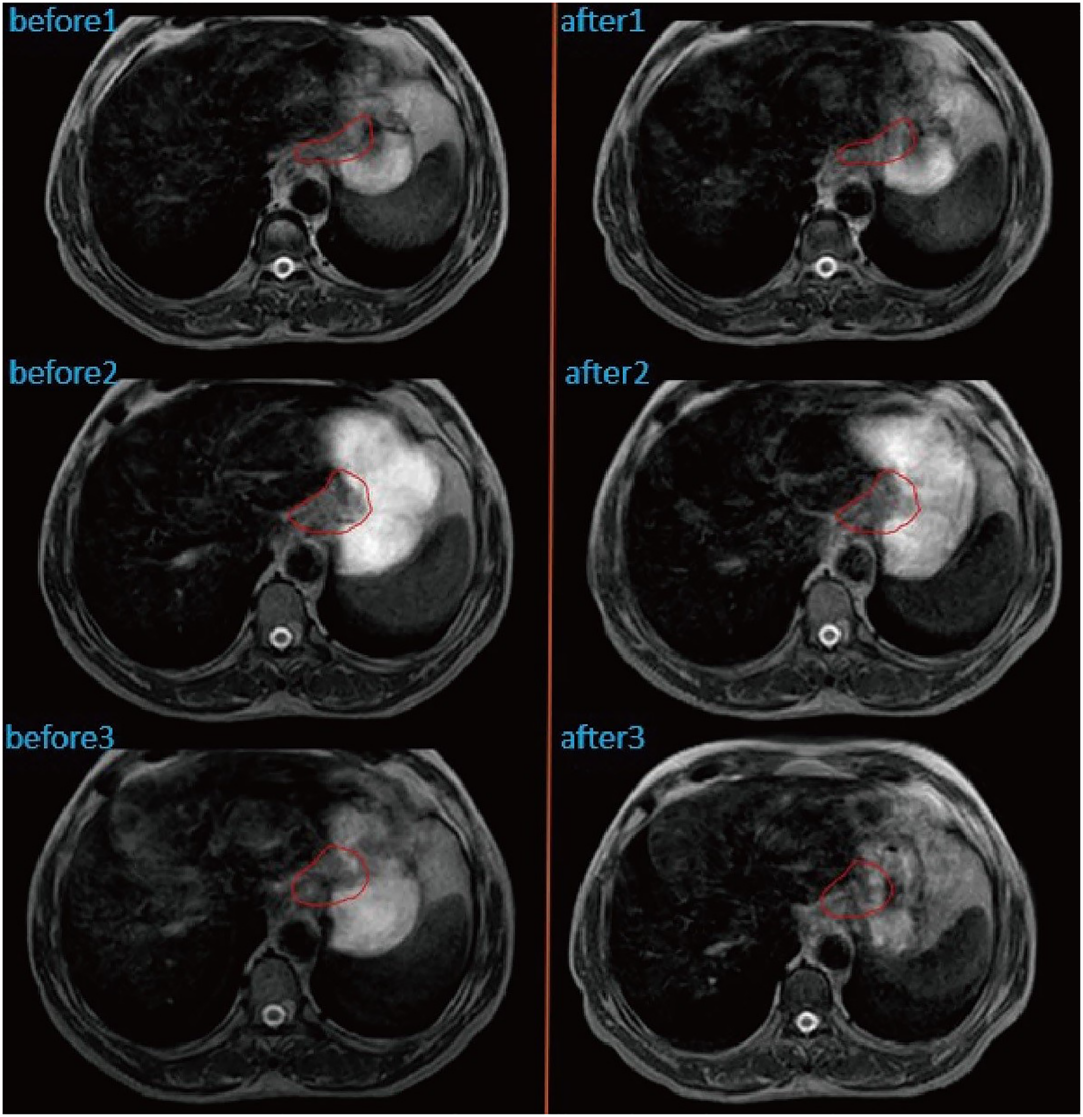
Intra-fractional changes of the anatomical structures by performing MRI before and after treatment at three different fractions. GTV was outlined in red.

Patient with a DSC of 0 at the second treatment, indicating the complete separation from the target area (**Figure 3**). The two target areas are unrelated, resulting in a significant separation of the target areas. Intra-segmental changes during the patient’s radiation treatment remained stable on both MRI imaging devices before and after treatment (**Figure 4**).

## Discussion and review of the literature

It is generally accepted that local tumor growth in malignant upper abdominal tumors is associated with severe symptoms and significantly reduced quality of life. As advances in systemic therapy have improved patient survival, effective treatments aimed at local tumor control and symptom relief are becoming increasingly important to ensure patient survival and a good quality of life. For this reason, radiation therapy as a minimally invasive local ablation therapy has sparked interest in the treatment of various tumors in the upper abdomen (8). The stomach is a non-static structure that changes significantly from day to day, taking into account changes in gastric volume, respiratory motion, and patient adjustment errors. Automatic segmentation of challenging organ contours such as the gastrointestinal tract remains controversial clinically (9). The patient in that article was scheduled to have the margin of the target volume (PTV) increased to 5 mm, which would result in an increase in the volume receiving irradiation. A margin of 5 mm from CTV to PTV would probably be a too narrow margin, in a case like this, if treated with conventional CB-CT based IGRT. Although an outward expansion of 5 mm was used, the later tumor location was not completely covered according to the CT simulation positioning (**Figure 2**). During the course of treatment, there were even instances where a patient received radiotherapy at one time with a planned target area that did not cover any of the lesions on that day (**Figure 3**). However, increasing the GTV dose in the presence of a larger PTV margin resulted in increased toxicity as the dose in the surrounding OAR. Therefore, high-precision radiation therapy is required to increase the dose to the tumor (10).

Introduction of intensity-modulated radiation therapy, advanced motion management solutions (e.g., respiratory gating), and image-guided radiation therapy (IGRT) techniques (e.g., CBCT) help reduce gastrointestinal side effects and increase dose to target volume to achieve higher bioequivalent doses (11). IGRT has become the routine standard of modern radiation therapy. Pre-treatment patient imaging can reduce the effects of configuration errors and improve the accuracy of dose delivery.

Although CBCT is very effective, it provides poor image quality for soft tissues, which makes accurate identification of soft tissue targets and surrounding OARs very difficult.

The higher morphological quality of MR images allows better visualization and design of treatment volumes compared to standard localization imaging (12). Emerging MRI-guided radiation therapy systems, such as the Elekta Unity system, allow continuous, real-time MRI guidance throughout the treatment. The superior image quality of MRI compared to CBCT allows visualizing the dynamic change of tumors daily. Furthermore, the concept of online MR-guided radiation therapy involves daily adjustment of the treatment plan to the current anatomy, thereby reducing the risk to deliver unwanted dose to the OAR (6). When a patient breathes, swallows or digests food, a patient’s internal organs move, and even the slightest movement can affect the location of the tumor, making precise radiation therapy difficult. MRI-LINAC uses continuous MRI to capture multiple frames per second to observe the motion of soft tissues and organs, and then compensate for these motions during treatment (13). However, the total treatment time using MR-LINAC with online adaptive planning is much longer than the conventional treatment method. Patients may have gastrointestinal motility effects during treatment. Therefore, we reduce gastrointestinal motility based on the patient’s scopolamine (10 mg) before each radiotherapy. The patient’s gastrointestinal motility stabilized before and after the split treatment (**Figure 4**).

As online MR guided adaptive radiotherapy is a relatively new technology, its unique features have shown that it can effectively deal with the daily geometric deformation of the target and surrounding OARs (14). It has been proved in a variety of upper abdominal tumors (8), prostate cancer (15) and rectal cancer (16). And the case has been reported in peritoneal carcinomatosis MRgRT with online adaptation method is technically and clinically feasible with clean toxicity result (17). Daily-adaptive MR-guided SBRT reported a significantly improved single intestinal loop sparing for lymph-nodal oligometastases. Also, bowel Dmax was significantly reduced with daily-adaptive strategy (18). However, reports on gastric cancer were rarely presented. Our clinical experience using MR-LIINAC has shown that using online MRgRT has a great potential to more effectively treat gastric cancer.

Our case showed that giving patients scopolamine before treatment session helped to reduce gastric peristalsis, resulting in clearer imaging. MRI-based treatment systems enable real-time tracking of these structures during radiation treatment, thus avoiding under-dosing of the tumor as well as over-dosing of key structures. We have shown that it is feasible to use on-line adaptive MR-guided radiotherapy for patients with gastric malignancies. However, the survival of patients with gastric cancer treated with MR-LINAC compared to conventional radiotherapy needs to be observed over time. Large-scale controlled studies are also needed to confirm the benefits of receiving MR-LINAC.

## Data availability statement

The original contributions presented in the study are included in the article/**Supplementary Material**. Further inquiries can be directed to the corresponding authors.

## Ethics statement

The studies were conducted in accordance with the local legislation and institutional requirements. The participants provided their written informed consent to participate in this study. Written informed consent was obtained from the individual(s) for the publication of any potentially identifiable images or data included in this article.

## Author contributions

YS wrote the first draft of the manuscript. YZ, HW, MZ, and FG wrote sections of the manuscript. YS, ZL, and JY managed the radiotherapy treatment plan processing and data analysis. ZL and JY contributed to image review and contouring. YS performed dose accumulation and generated figures. All authors contributed to manuscript revision, read, and approved the submitted version.
