# Supplementary material for: Case Report: MR-LINAC-guided adaptive radiotherapy for gastric cancer
Source: Front Oncol. 2023 Sep 8;13:1159197. doi: 10.3389/fonc.2023.1159197 (PMC10514477; doi:10.3389/fonc.2023.1159197)
Supplement: Supplementary file 1 [file DataSheet_1.docx]

Supplementary Material

**Supplementary Figures**


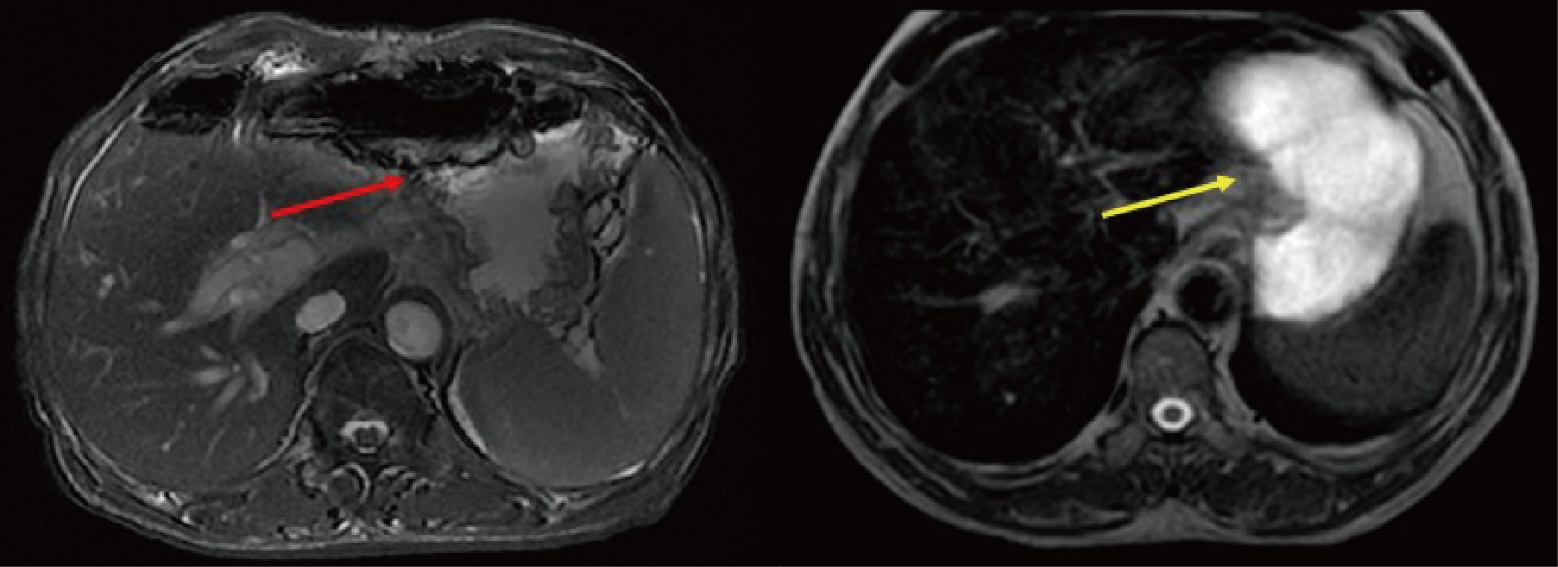


**（B）**

**（A）**

Figure S1. Comparison of stomach contours. (A)Magnetic resonance image (MRI) of a patient (not the patient treated in this study) not treated with anisodamine (red arrow); (B) MR image of the patient treated with anisodamine; notice that the outline of the stomach is clearer (yellow arrow).


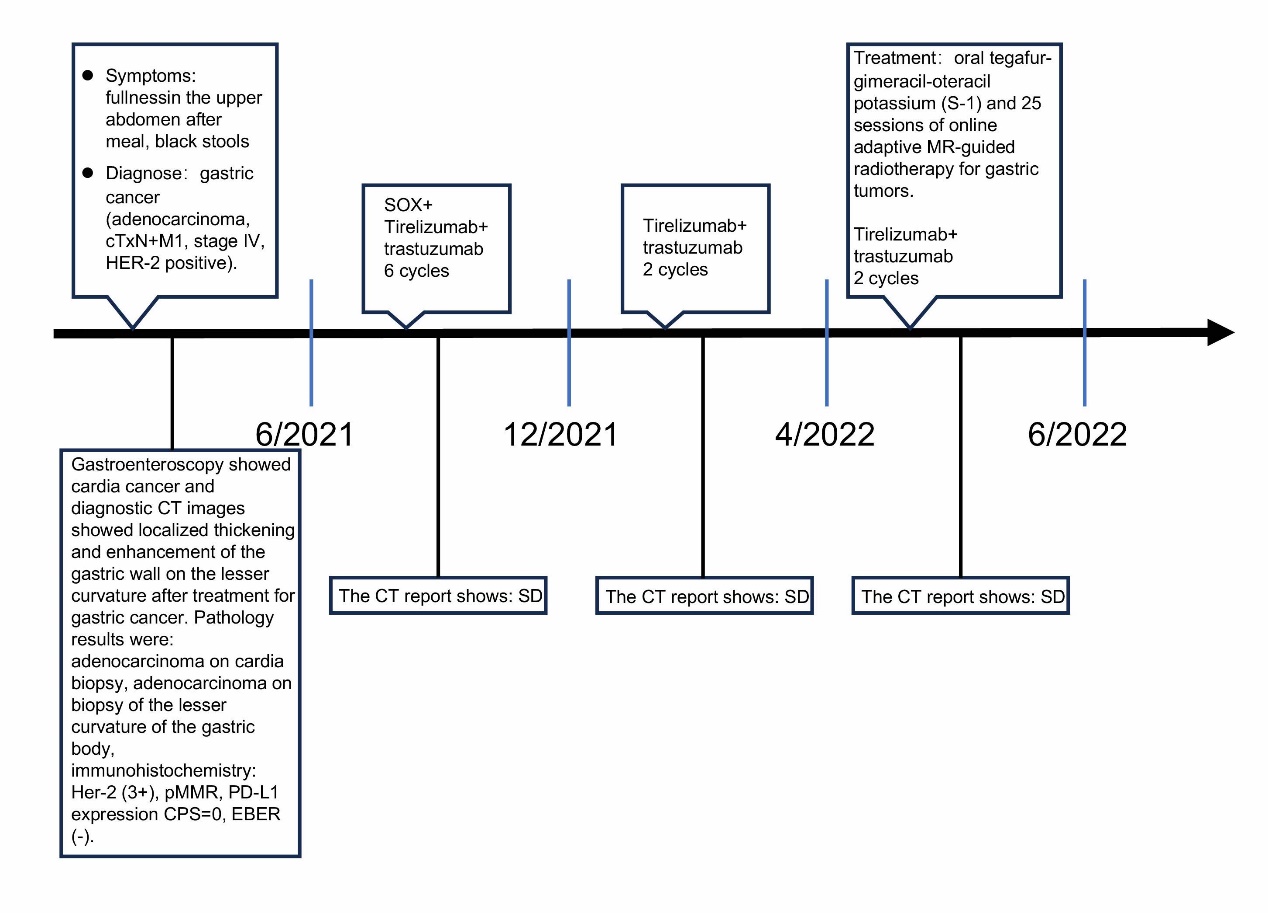


Figure S2. Timeline of interventions and outcomes. SOX: tegafur-gimeracil-oteracil potassium (S-1) + oxaliplatin. SD：stable disease.
